# Genetic population structure and reproductive system of two invasive Asian earthworms, *Amynthas tokioensis* and *Amynthas agrestis*

**DOI:** 10.7717/peerj.13622

**Published:** 2022-07-13

**Authors:** Maryam Nouri-Aiin, Samantha Connolly, Cheryl Keough, Annie Jean Smigelsky, Yiyi Wen, Jeremy Howland, Jos. J. Schall, Josef H. Görres

**Affiliations:** 1 Plant and Soil Science Department, University of Vermont, Burlington, VT, United States of America; 2 Plant Biology Department, University of Vermont, Burlington, VT, United States of America; 3 Environmental Science, Rubinstein School of the Environment, University of Vermont, Burlington, VT, United States of America; 4 Department of Biology, University of Vermont, Burlington, VT, United States of America

**Keywords:** *Amynthas*, Micorsatellite markers, Genetic diversity, Ploidy level, Clonality, Reproductive system, Population genetic structure, Jumping worms, Invasive earthworms

## Abstract

The invasive Asian earthworms, *Amynthas tokioensis* and *A. agrestis*, have been successful in entering North American forests in recent decades, with significant damage to both soils and above-ground environments. This success could be driven in part by a polyploid genetic system and parthenogenetic reproduction, often suggested as benefits for invasive species. Therefore, we assessed the genetic population structure, genetic diversity, and reproductive system of both species using morphological traits and panels of microsatellite markers. A total of 216 *A. tokioensis* and 196 *A. agrestis* from six sites in Vermont USA were analyzed. Although all worms were morphologically hermaphroditic, all the *A. agrestis* lacked the male pore (the structure allowing pass of sperm between individuals), and only 19% of the *A. tokioensis* possessed the male pore. All *A. tokioensis* earthworms were triploid (scored for three alleles for at least 1 locus, and usually several), and *A. agrestis* was a mix of triploid and diploid individuals. Notable was the high proportion (80%) of *A. agrestis* earthworms that were diploid at one site. There was clearly clonal reproduction, with identical seven- locus genotypes observed for earthworms from each site, with as many as 45 individuals with the identical genotype at one site. However, the earthworms were also genetically diverse, with 14 genotypes observed for *A. tokioensis* and 54 for *A. agrestis*, and with many singleton genotypes (a single individual). Most genotypes (71% for *A. tokioensis* and 92% for *A. agrestis*) were found at a single site. The greatest number of genotypes was found at a commercial nursery where fully 23/26 *A. agrestis* earthworms were singleton genotypes. As expected for the pattern of private clone alleles at sites, several measures of geographic genetic differentiation were positive, and as expected for triploid systems, an AMOVA analysis showed high within-individual genetic diversity. The paradox of clear clonal reproduction, but with a great number of genotypes for each species, and the mix of triploid and diploid individuals could be explained if the worms have been sexually reproductive, with the switch to the uniparental system only recently (or even if sexual reproduction is episodic). Last, a large number of microsatellite loci were recovered for each species and there sequence and suggested PCR primers are provided for free use by other researchers.

## Introduction

Invasive species, ranging from microbes (including spill-over of pathogens into naïve hosts) to large organisms such as trees and vertebrate animals, result in enormous economic cost every year, with fully a 125 billion dollars loss in the USA alone (Allendorf & Lundquist, 2003). More permanent is damage to natural habitats and biodiversity reduction. Such movement of species across geographic regions, by gradual migration or sudden long-distance shifts, is the very nature of life on earth. Thus, invasive species are part of an ongoing, unwelcome, experiment in biogeography. Alfred Wallace, founder of the modern study of biogeography, was particularly concerned with the question of how organisms that move to a new area are able to thrive in their adopted environments and compete within local, well adapted, communities of other species. His magisterial work on island biogeography highlighted this question for long-distance migrants (Wallace, 1876), and presaged the question of how introduced species can be successful in new environments. Sakai et al. (2001) list the characteristics of invasive species that may play a role in their success (although those also often apply to species that are long-term endemics). Broadly these include behavioral traits (such as broad diet), physiological tolerance, life history traits including parthenogenesis and short generation time, and dispersal modes. Although many ecological challenges are faced by an incoming species (such as an initial small propagule size), the traveler would also suffer a founder effect, and likely genetic bottleneck and loss of genetic diversity (Allendorf & Lundquist, 2003). Propagule pressure could be substantial, of course, if there are multiple invasion events with a large number of individuals (Novo et al., 2015; Keller et al., 2020). Allendorf & Lundquist (2003) note that invasion biology opens a window into evolution within small populations and when a species enters a new environment requiring local adaptation, perhaps at an accelerated rate.

A recurring idea is that parthenogenesis and polyploidy would aid traveler species (Edwards & Bohlen, 1996; Díaz Cosín, Novo & Fernández, 2011). With parthenogenesis only a few individuals could act as the invading propagule, and the unisexual reproductive system would eliminate inbreeding in a small population and also result in rapid population growth. Parthenogenesis could be linked to polyploidy which would allow a high within-individual genetic diversity, the *F*_is_ of population genetics (Freeland & Kirk, 2011). Terhivuo & Saura (2006) propose that polyploidy would also provide more targets for mutation, and thus accumulation of genetic diversity even in a parthenogenetic species. Polyploidy would also be adaptive if there is an “all purpose” genotype that would meet new environmental conditions (Baker, 1965; Díaz Cosín, Novo & Fernández, 2011; Fernández et al., 2011). Note that some of these speculations could be reversed such that parthenogenesis would reduce overall genetic diversity in a population (no sexual recombination) and polyploidy could simply hide deleterious recessive mutations.

**Figure 1:**
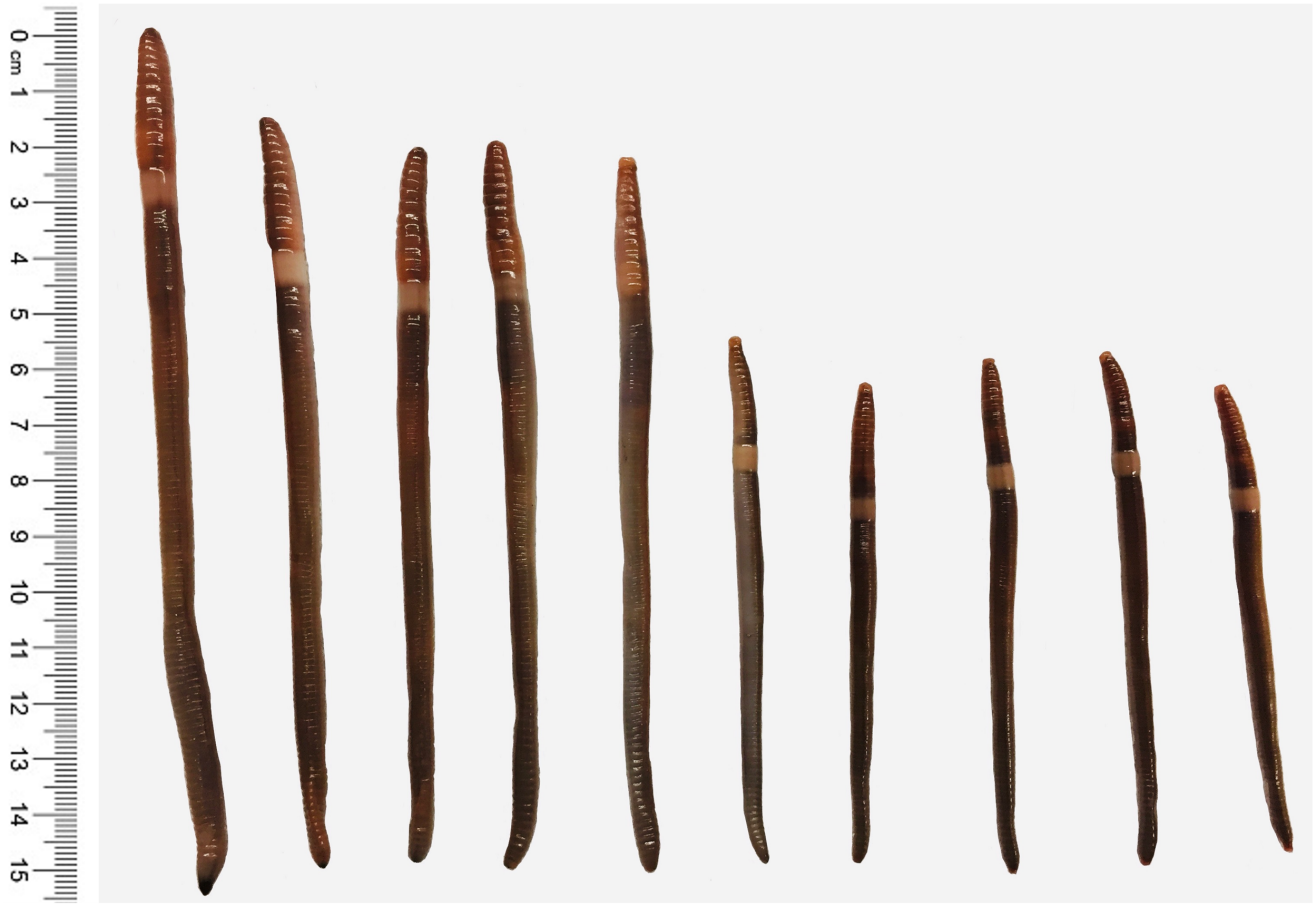
Two understudied *Amynthas* species. *Amynthas tokioensis* (five earthworms in the right) and *Amynthas agrestis* (five earthworms in the left).

**Figure 2:**
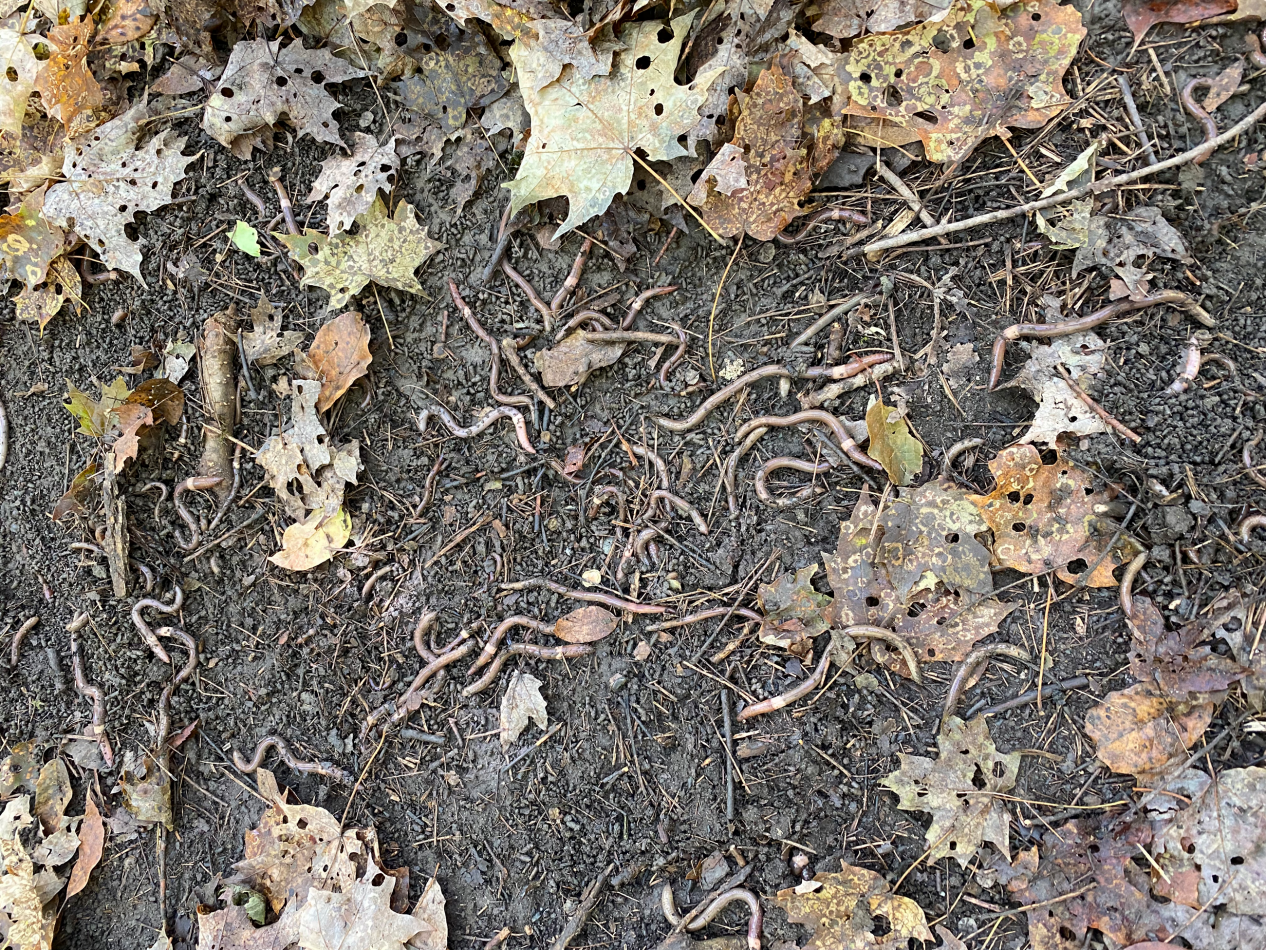
*Amynthas* infestation. High density of *Amynthas* individulas in Montpelier, VT.

We focus on these issues with a study of the genetic diversity and reproductive system of two important invasive earthworms, *Amynthas tokioensis* (Beddard, 1982) and *Amynthas agrestis* (Goto and Hatai, 1899) (Oligochaeta, Megascolecidae) in New England, USA (Fig. 1). These earthworms have been notably successful invasive species, with a broad geographic range (Chang et al., 2021), and locally can be found in very high dense populations in soils (Fig. 2). After the Pleistocene glacier retreat in central and northern North America ∼10,000 ya, soil communities, including earthworms, are assumed to have been eliminated (Edwards & Bohlen, 1996; Terhivuo & Saura, 2006). Movement of native North American earthworms appears to have been very slow (∼1–9 m yr^−1^), and only a few native earthworms are known from areas north of the southern limit of the glaciation or perhaps the southern limit of otherwise frozen soils (Edwards & Bohlen, 1996; Díaz Cosín, Novo & Fernández, 2011). Thus, almost all of the earthworms now found in those regions were introduced, first being invasive, and then naturalizing in the soil community. The first wave were ∼30 species from Europe, brought by colonizers, beginning about 300 years ago (Hendrix et al., 2008). More recent is the “second wave” of invasive earthworms from Asia, primarily peregrine species in the Pheretimoid clade (12 genera within the Megascolecidae; Chang et al., 2021). Now these earthworms have invaded fully 38 US states, and several locations in Ontario province, Canada (Moore, Görres & Reynolds, 2018; McCay et al., 2020; Reynolds & Mctavish, 2021; Moore, Ouimet & Reynolds, 2022). Included are several *Amynthas* species that originated from Japan (Nouri-Aiin et al., 2021; Blakemore, 2003). *Amynthas* are known to cause serious disruption of the invaded soils, including chemical and physical alteration, and the elimination of resident earthworms and other invertebrates (Snyder, Callaham & Hendrix, 2011; Greiner, Kashian & Tiegs, 2012; Laushman, Hotchkiss & Herrick, 2018). The disruption of soil environments by these earthworms should also have serious consequences for above-ground communities, and even ecosystems driven by complex ecological cascades (Hale, Frelich & Reich, 2005; Callaham et al., 2006; Frelich et al., 2006; Frelich et al., 2019; Hendrix et al., 2006; Bardgett & van der Putten, 2014).

Our goal is to cast light on the characteristics of *Amynthas* earthworms that could allow them to invade and become established in soils where the earlier introduced species from Europe have long been present, presumably adapted, and now naturalized. There are hundreds of species of *Amynthas* widely distributed in Asia, and 16 have been introduced to North America, yet only three are considered to be widely invasive. Several characteristics of the invasive *Amynthas* earthworms have been proposed to aid entry into established soil communities, including a broad diet (Zhang et al., 2010), tolerance of their eggs and embryos within a covering cocoon to drought and cold (Görres et al., 2018), and suspected parthenogenesis and polyploidy (Hendrix et al., 2008; Minamiya et al., 2011). Earthworms are typically hermaphrodites, but both *A. tokioensis* and *A. agrestis* often lack some of the male reproductive structures including spermathecal pores, spermathecae, male pores, prostate glands, and their associated genital markings (Chang, Snyder & Szlavecz, 2016), which strongly suggests they have abandoned the male function and reproduce parthenogenetically (Chang et al., 2021). Identifying these species morphologically requires examination of the male parts, *i.e.*, characters often missing, so rapid molecular methods have recently been developed (Nouri-Aiin & Görres, 2021).

Two studies hint that *Amynthas* species are often parthenogenetic and polyploid. Shen et al. (2011) used flow cytometry and karyology to show that *Amynthas catenus* (Tsai, Shen, Tsai, 2001) in Taiwan is a mix of 2N, 3N, and 4N individuals, and Cunha et al. (2017) used a panel of microsatellite markers and a small number of *Amynthas corticis* (Kingberg, 1866) from an Azores island to find that all of the earthworms were 3N for at least one locus. Terhivuo & Saura (2006) note that chromosomal sex determination may be a barrier to evolution of polyploidy in animals, but earthworms lack this mechanism of sex determination so the switch to polyploidy is an option.

Because of their diverse reproductive systems, earthworms provide a useful system to explore the evolution of sex (Jaenike & Selander, 1979; Jaenike, Ausubel & Grimaldi, 1982; De Sosa et al., 2017; Díaz Cosín, Novo & Fernández, 2011). Most species are both morphologically and functionally hermaphroditic; that is, most are biparental sexually reproductive. Many other species reproduce uniparentally, but retain at least some male structures. Jaenike & Selander (1979) developed a model, and tested it with studies on *Octolasion tyrtaeum* and *O. cyaneum* to show that under some situations there is a transition between sexual and parthenogenetic reproductive systems, and diploidy to polyploidy. Further, Jaenike, Parker Jr & Selander (1980) found that parthenogenetic earthworms can have a broader habitat use than sexually reproducing earthworms; this could be a result of heterosis and broad environmental tolerance of polyploids.

In our study of the two invasive *Amynthas*, we used microsatellite loci, with codominant alleles to seek information on the genetic structure and reproductive system of the earthworms. Our goals were, first, to examine the overall genetic diversity of the two species. If the initial propagule of the invasion was small, and the worms are parthenogenetic, it may have led to low genetic diversity. If so, that begs the question of how they can be so successful. Second, we asked if there is any geographic genetic structure in the two earthworm species. This would lead to some insight into how the earthworms have been moved about across sites. We then sought to determine if the earthworms reproduce by parthenogenesis by finding of clones of genetically-identical earthworms. Last, the results would show if the two earthworm species are polyploid. The model of Jaenike and colleagues of a transition in the reproductive system suggests an initial mixture of ploidy levels. This is the first such large-scale study on the *Amynthas* group using variable genetic markers, or indeed for any species in the Megascolecidae.

## Materials and Methods

### Earthworm specimen collection

**Figure 3:**
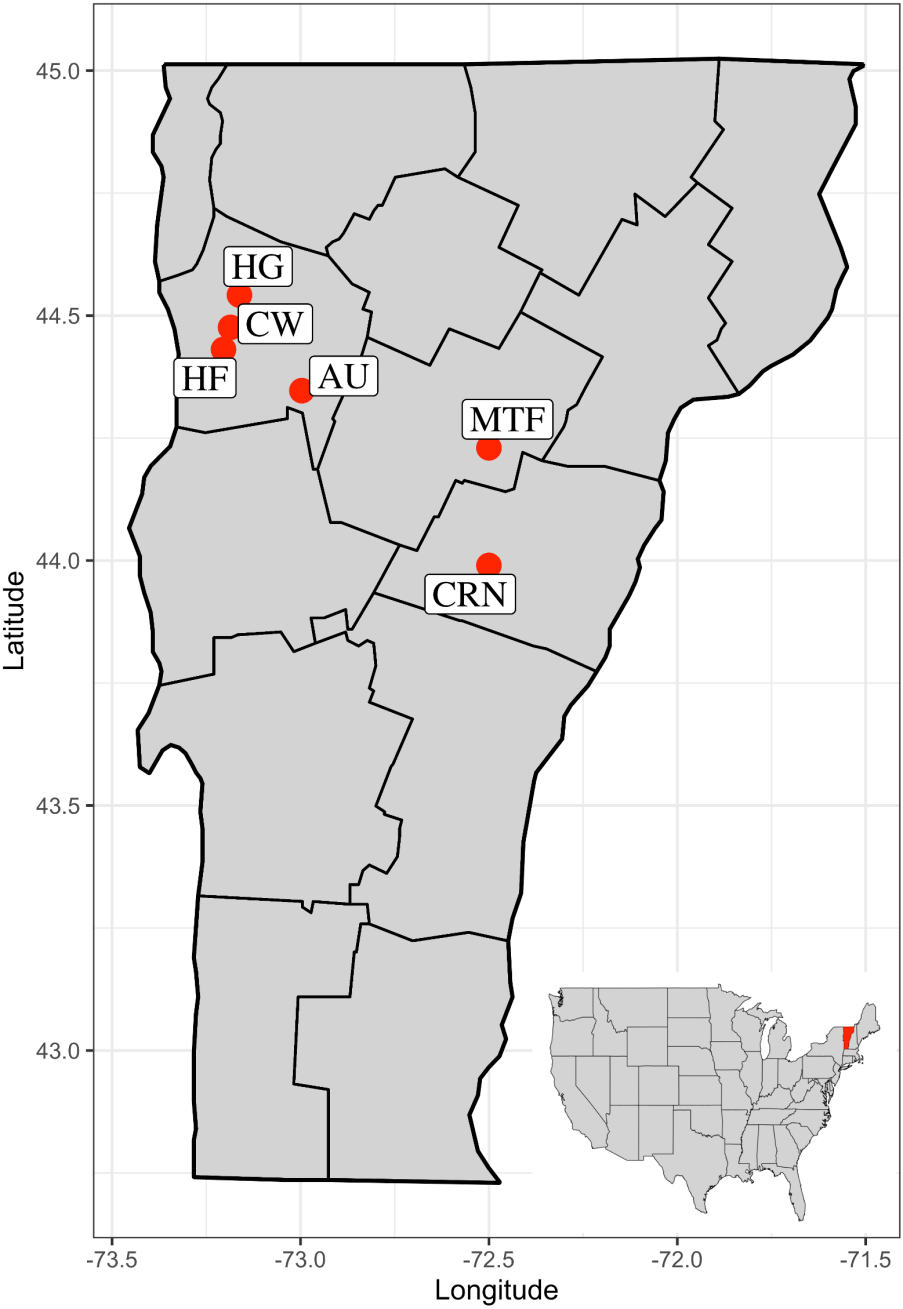
Map of the state of Vermont, USA, with county boundaries indicated. Six sites used in the study are Commercial, Retail Nursery (CRN), National Audubon Society Nature Preserve (AU), Centennial Woods Natural Area (CW), Municipal Tree Farm (MTF), Home Garden (HG), Horticultural Research and Education Center of University of Vermont (HF).

From May-August 2019, we collected specimens of *Amynthas tokioensis* and *A. agrestis*, from six sites in Vermont, USA (location details, site code, and sample sizes are shown in Table 1). These sites were chosen to include a range of habitat, soil types, and disturbance history: an ornamental plant nursery, a municipal tree farm, a home garden, and three natural areas. The sites range from 7 km to 144 km distant from one another (Fig. 3). The earthworms were collected by manually turning the upper 15 cm of litter and soil where the earthworms live (thus, an epi-endogeic earthworm habitat). The earthworms were taken to the laboratory, where they were examined for presence of male reproductive structures (spermathecal pores, spermathecae, male pores, prostate glands, and their associated genital markings). *Amynthas* earthworm juveniles are often impossible to identify to species by morphology, so to identify the individuals and to insure only the two target species were present in the study (cryptic species may be common in earthworms; (Novo et al., 2010), we used a species-specific PCR multiplex method for identification. Briefly, a panel of ∼100 individuals were sequenced for the mitochondrial COI barcoding gene (Hebert, Ratnasingham & De Waard, 2003) that revealed only the two species were present at the sites, then another group of 40 individuals that could be unambiguously identified by morphology were sequenced for the COI gene, and species-specific primers that produced different sized amplicons were designed. This method was then tested and gave clear unambiguous identification for the two species (details in Nouri-Aiin & Görres, 2021). The adult earthworms could often be identified by morphology (Chang, Snyder & Szlavecz, 2016), but sometimes, due to various degree of degradation in external and internal characteristics associated with their reproductive organs, identification required the barcoding method. The earthworms were taken to the laboratory where they were washed in RO-H_2_O, killed in 50% ethanol, washed again, and dried. We removed the anterior 4 segments and stored them in individual 1.5 ml vials at −20 °C.

**Table 1:** Samples information. Sampling sites with site code, GPS coordinates, and sample sizes for two species of invasive earthworms, *Amynthas tokioensis* and *Amynthas agrestis*. Location of the two private properties are given as approximations to provide privacy.

Sites and site codes	Latitude, Longitude Elevation (m)	Sample size	
		*A. tokioensis*	*A. agrestis*
Commercial, Retail Nursery (CRN)^∗^	43.9, −72.5, 199	37	26
National Audubon Society Nature Preserve (AU)	44.32, −72.98 196	33	14
Centennial Woods Natural Area (CW)	44.47, 73.18 83	5	45
Municipal Tree Farm (MTF)^∗^	44.23, −72.50 338	29	25
Home Garden (HG)	44.5, −73.2 59	27	22
Horticultural Research Center of University of Vermont (HF)	44.42, −73.21 48	85	64
Total		216	196

### Microsatellite library development and marker selection

DNA was extracted from a single individual of each earthworm species taken at the HF site using the E.Z.N.A. Tissue DNA kit (Omega Bio-Tek, Norcross, GA, USA) following the supplier’s protocol, and submitted to the Cornell University Evolutionary Genetics Core Facility (Ithaca, New York, USA). There a simple sequence repeat (SSR) enriched library was constructed. Using the method of Hamilton et al. (1999) genomic DNA was restriction digested and enriched for microsatellites with a panel of 14 probes with a variety of repeat motifs. Fragments were then sequenced using the Titanium 454 platform (454 Life Sciences, Branford, CN). The result was a library of ∼33 ×10^3^ fragments for *A. agrestis* and ∼26 ×10^3^ for *A. tokioensis*. The full set of fragments, their length, repeat motif, and suggested PCR primers and programs is given in Supplemental Information 1 and 2 for free use by researchers.

Markers were selected with 3–6 bp repeats because they would likely be easier to score on standard fragment analysis pherograms, and with longer repeats because these were more likely to be variable. We then tested a panel of fragments (44 for *A. agrestis*, 36 for *A. tokioensis*) using PCR with labeled primers (details below). We sought fragments that amplified without failure (reducing likely null alleles), and that produced clear, unambiguous peaks on electropherograms, and with minimal stutter. Of successful candidates we selected those that were polymorphic, resulting in a panel of 7 markers for each earthworm species (Table 2). This selection process led to overall high-quality results; that is, PCR amplification was always successful, and pherograms throughout the study were clear and straight-forward to score.

**Table 2:** Microsatellite loci characteristics. Microsatellite markers for two species of invasive earthworms, *Amynthas tokioensis* and *Amynthas agrestis*, giving the locus identification code, repeat motif, primers used, product size range in bp, and PCR program.

Locus code	Repeat motif	Primer sequence (5′ − 3′)	Product size (bp)	Genbank accession number	PCR program
*Amynthas tokioensis*					
8188	(AAGT)_25_	F: CATGCCTAGAGAGAATAAGCAGC R: TGAGCATGAGTTGAGAGTTTGAG	228–248	OM648925	Program 1
906	(AAGT)_16_	F: CAAAGGAAGGAAAGACGGTAACC R: GAGTGCAGTCCATTCCATTAGTTG	174–250	OM648919	Program 1
943	(AAGT)_28_	F: CTGTTAAGTGTTCAGTGTTCCCG R: TTCGTATTCAAGAGTTTCGCGAG	269–380	OM648920	Program 1
1053	(ATC)_22_	F: AACCTCTTCAAGACCTATGGACC R: TTGGTGATCGCTACCAATGAGAG	169–214	OM648923	Program 1
1176	(ACAT)_13_	F: GCATTAACTGTGTGACATGCTTG R: CAGTTAAAGATTTGTTGACCTGCC	182–314	OM648924	Program 1
1011	(AAGT)_13_	F: CTACTGCGGTACTTCTTTCCAAG R: GAATTCTTAGTCGCGTACAGTCC	206–234	OM648922	Program 1
984	(AAGT)_11_	F: ATGCAATGTACTGAAGTGCTAGC R: AATGAATTTGTGTCGTCTGTCCG	166–182	OM648921	Program 1
***Amynthas agrestis***					
5016	(AAGT)_28_	F: ATAAATGACCCTGCACGAATGAC R: AATACATTACTTTCGTCGTCGCTGAC	260–360	OM648929	Program 1
3786	(ACT)_8_	F: TCTGCACTACTACATTGTACTGTG R: GCGCTAAAGAAGTCTACTGCTAC	207–225	OM648928	Program 1
9389	(ACT)_8_	F: CAGCGAACGGATCTCACAAG R: TCTGAATACATGAATGGGTTTGTTG	207–267	OM648931	Program 2
13732	(AGAT)_16_	F: TCTTGCAGATGTCAATGATCACG R: AGCAGTTTGTTAGACCACATAATTC	216–262	OM648932	Program 2
652	(TCTA)_26_	F: CTCTTGGTGTGATCATGTGACTC R: ACTACGTGATCAAACCTTATCGC	200–300	OM648927	Program 1
6034	(ACAG)_27_	F: TGGATGAAAGGGAATGTTCGTTG R: TAAACTGAAGAACTGCATCCACG	172–326	OM648930	Program 1
628	(ACAT)_25_	F: TGACTCTCTAATAATGCGCTTGTTAG R: GTAGAACGACGGACTCTGAGATC	230–254	OM648926	Program 2

### Molecular methods

Frozen tissue for each earthworm was brought to room temperature, the DNA was extracted using the E.Z.N.A. kit as described above, and DNA stored at 4 °C. Each sample DNA was then PCR amplified (TopTaq PCR kit, Qiagen, Hilden, Germany) using primers presented in Table 2, with the forward primers 6FAM dye labeled (Integrated DNA Technology, Coralville, IA). Two PCR programs were used in the study: PCR programs 1: 94 °C (4 min), 32 cycles of [94 °C (50 s); 58 °C (30 s); 72 °C (1 min)], with a final extension of 10 min at 72 °C, and PCR program 2: 94 °C (4 min), 30 cycles of [94 °C (50 s); 56 °C (30 s); 72 °C (1 min)], 8 cycles of [94 ° C (30 s); 54 °C (30 s); 72 °C (1 min)] with a final extension of 10 min at 72 °C. The product was then checked on a 1% agarose gel, to determine an appropriate dilution, and added with a LIZ500 size standard into Hi-Di formamide (Life Technologies, Foster City, CA). Samples were then processed at the Cornell University Core Laboratory (Ithaca, NY) on the 3730xL Genetic Analyzer (ThermoFisher Scientific, Wilmington, DE).

**Table 3:** Indices of genetic diversity for *Amynthas tokioensis* and *Amynthas agrestis*. Panel (A) Data for each locus across sites. Given are locus codes from, number of alleles (Na), observed heterozygosity (Ho), expected heterozygosity (He), and the inbreeding coefficient (G_*is*_). G_*is*_ compares Ho and He, with values of zero when the frequency of heterozygous equal the Hardy-Weinberg expectations, 1.0 when there are no heterozygotes, and −1 when there is an excess of heterozygotes. Values deviating from zero indicated inbreeding (or clonal reproduction which is equivalent to extreme inbreeding). Maximum likelihood method was used to correct for the unknown dosage of the alleles, that is polyploidy. Panel (B) Data for each site across loci. Mean of number alleles across all loci (Na mean), observed heterozygosity (Ho), expected heterozygosity (He), and inbreeding coefficient (G_*is*_) as described for (A) (Site codes: Commercial, Retail Nursery (CRN), National Audubon Society Nature Preserve (AU), Centennial Woods Natural Area (CW), Municipal Tree Farm (MTF), Home Garden (HG), Horticultural Research and Education Center of University of Vermont (HF)).

Panel A				
*A. tokioensis*				
Locus	Na	Ho	He	G_is_
8188	2	0.00	0.24	1.00
906	11	0.96	0.73	−0.38
943	7	0.70	0.61	−0.25
1053	7	0.60	0.6	0.00
1176	5	0.87	0.67	−0.43
1011	3	0.58	0.50	−0.28
984	4	0.12	0.58	0.76
*A. agrestis*				
5016	12	0.19	0.30	0.23
3786	5	0.00	0.14	1.00
9389	11	0.10	0.78	0.79
13732	7	0.03	0.46	0.91
652	21	0.97	0.87	−0.28
6034	17	0.58	0.75	0.06
628	7	0.44	0.82	0.32
Panel (B)				
*A. tokioensis*				
Populations	Mean Na	Ho	He	G_is_
CRN	4.14	0.43	0.51	0.16
AU	2.43	0.57	0.49	−0.17
CW	2.14	0.57	0.54	−0.05
MTF	2.57	0.57	0.48	−0.19
HG	2.00	0.57	0.41	−0.34
HF	3.43	0.57	0.48	−0.18
*A. agrestis*				
CRN	8.7	0.49	0.65	0.24
AU	4.3	0.21	0.62	0.64
CW	1.7	0.33	0.24	−0.39
MTF	1.7	0.32	0.31	−0.05
HG	2.9	0.31	0.48	0.34
HF	5.3	0.26	0.38	0.35

### Analysis

We scored alleles by visual examination of resulting pherograms using Geneious Prime 2021.2.2 (Biomatters, Aukland, New Zealand). Measures of allele numbers, allele frequency, genetic diversity, and clonal lineage composition were analyzed using the GenoDive program (Meirmans, 2020). We originally suspected that the earthworms are polyploid (which was confirmed by our analysis below), so for most of the analysis, ploidy-independent methods were used (Meirmans & Van Tienderen, 2004). The GenoDive program corrects for an unknown allele dosage via a Maximum Likelihood method. Genetic structure across sites was determined with GenoDive for *F*_st_ and Nei Distance measures, and AMOVA to partition genetic variance into individual, population, and among site contributions (Meirmans, 2020). To determine any genetic distinction in the worms among sites, a Principal Coordinates Analysis (PCoA) (Gower, 1967), was used to generate a matrix of pairwise genetic distances between individuals to produce a plot of the first two principle coordinates using Polysat 2021.09.1+372 R package (Clark & Schreier, 2017). Two measures of genetic distance were used (Bruvo et al., 2004; Lynch, 1990) in the PCoA. Bruvo’s method takes mutational distance (repeat distance in the microsatellite allele) into account and the Lynch’s method uses a simple band-sharing measure. This is analogous to the R_st_ vs. *F*_st_ of population genetics (Clark & Schreier, 2017). This method yields a graph showing worms falling into clusters of lowest genetic distance, that are recognized by eye, but not statistical significance.

## Results

### Summary statistics on allelic variation

Genotyping results were conducted for seven loci for each earthworm species for 216 *Amynthas tokioensis* and 209 *A. agrestis* (Table 3). Across all loci, the number of alleles varied from 2–11 for *A. tokioensis* and 5–21 for *A. agrestis*, which is similar to results for other earthworm species (Keller et al., 2020; Fang et al., 2021). Overall genetic diversity was high, with observed heterozygosity values (Ho) >0.90 for some loci for both species. However, expected heterozygosity (based on frequency of alleles and their random association) and observed heterozygosity values differed for some loci across sites, and within sites across loci (that is, the population is not in Hardy–Weinberg expectations). The G_is_ is a measure of inbreeding based on a comparison of these two values, and is zero when heterozygosity is in Hardy–Weinberg expectations, 1.0 when there are no heterozygotes, and −1 when there is an excess of heterozygotes (Nei, 1978; Nei, 1987). A striking result is that G_is_ values could be both strongly positive (meaning a deficit of heterozygotes) or moderately negative (a surplus of heterozygotes) compared to random association of alleles. This outcome is expected when parthenogenesis leads to multiple clones of genetically-identical individuals.

### Evidence for triploidy and clonal reproduction

Examination of pherograms found that earthworms presented either two or three peaks (Table 4). If an earthworm presented three peaks for at least one locus, and more often 2 or more loci, it was scored as triploid. In contrast, if all loci presented one or two peaks, that earthworm was scored as a possible diploid, although there would be a very small chance it was triploid. All *A. tokioensis* were scored as triploid at all sites, and all *A. agrestis* were scored triploid at four sites. A striking anomaly in the results is that 80% of *A. agrestis* earthworms at HF showed only one or two peaks at every locus. The scoring protocol thus would conclude that most *A. agrestis* earthworms at HF were diploid. We assume that triploid earthworms are likely to be parthenogenetic; for hermaphroditic earthworms this would mean that the male function is absent. All adult *A. agrestis* (*N* = 196) lacked male pores, and 131/216 *A. tokioensis* were adults and only 25 had male pores and associated prostate glands. However, none of these were diploid. This suggests that the earthworms can be parthenogenetic even when diploid.

**Table 4:** Evidence for clonal reproduction in *Amynthas tokioensis* (Panel A) and *Amynthas agrestis* (Panel B). Nei’ corrected diversity index was used to assign clones. Presented are the number of earthworms sampled at each site (Sample N) with the number of 7-loci genotypes (a genotype is identical for all loci) (Genotype N) and number of genotypes found only at one site (Private genotypes). A “genotype can be a single worm or multiple worms with the same genotype. Possible ploidy levels is given as two (diploid) when one or two alleles are identified across all loci for an earthworm, or three (triploid) when three alleles are identified for at least one locus. “Evenness indicates how evenly the genotypes are divided over the population (an evenness value of 1 indicates that all genotypes have equal frequencies). “Evenness gives a measure of diversity independent of sample size (a version of the Simpsons diversity index). “Neis genetic diversity corrected known as Simpson’s diversity index present diversity independent of the sample size. Shannon index corrected for sample size is a version of the Shannon index that uses a non-parametric bias correction (Site codes: Commercial, Retail Nursery (CRN), National Audubon Society Nature Preserve (AU), Centennial Woods Natural Area (CW), Municipal Tree Farm (MTF), Home Garden (HG), Horticultural Research and Education Center of University of Vermont (HF)).

Site	Sample N	Genotype N	Private genotypes	Ploidy		Nei’s genetic diversity corrected	Evenness	Shannon index corrected
				Diploid	Triploid			
Panel A								
*A. tokioensis*								
CRN	37	6	2	0	37	0.52	0.34	0.46
AU	33	4	2	0	33	0.57	0.56	0.40
CW	5	2	0	0	5	0.60	0.96	0.29
MTF	29	3	1	0	29	0.58	0.76	0.41
HG	27	1	0	0	27	0.00	1.00	0.00
HF	85	7	5	0	85	0.54	0.32	0.43
Panel B								
*A. agrestis*								
CRN	26	23	23	0	26	0.98	0.77	1.32
AU	14	11	9	7	7	0.96	0.81	1.00
CW	45	1	0	0	45	0.00	1.00	0.00
MTF	25	2	1	0	25	0.45	0.89	0.27
HG	22	6	5	0	22	0.71	0.45	0.64
HF	64	15	12	51	13	0.73	0.23	0.78

Additional evidence for parthenogenetic reproduction comes from the presence of genetically-identical clones. At all sites, there were groups of genetically identical (across all loci) individuals, called here “genotypes”, ranging from genotype groups with two earthworms at several sites to a single genotype of *A*. *agrestis* (*N* = 45) sampled at CW and a single genotype of *A. tokioensis* (*N* = 27) at HG (Table 4). There were also many singleton genotypes; that is, a genotype were found in only a single earthworm. Each seven-loci genotype was assigned a code with 14 genotypes for *A. tokioensis* and 54 for *A. agrestis*, including genotypes found only once (Table S1). Some genotypes were found at multiple sites, with *A. tokioensis* genotype G1 found at all, and G3 at five sites. Most other genotypes were found only at a single site; thus, private genotypes were present, and sometimes common, at all sites. The commercial nursery (CRN) had the most diversity of genotypes for *A. agrestis*, of 26 earthworms sampled there, there were fully 23 unique genotypes. Thus, a large diversity of genotypes were identified despite the apparent clonal reproduction of both species.

In a sexually reproducing species, two individuals could reveal the same alleles at a locus by chance. This probability is low with as many as 21 alleles for a microsatellite locus (Table 3), and infinitesimally small across seven loci. When some genotypes were found as many as 45 times at a site for one species (*A. agrestis* at CW), this pattern could only result from clonal reproduction. Overall, we interpret these results as evidence of parthenogenetic reproduction leading to clones of earthworms for both species, with multiple clones found at most sites, and with some clones found at several of the sites. Although there was substantial genetic diversity in the earthworms of both species, that diversity was partitioned into clones of earthworms.

### Differentiation among sites

Several measures were examined to detect geographical population structure across sites. Two measures, *F*_st_ and Nei Genetic Distance, using standard allele frequency data and no consideration of clonal structure (Table 5). As expected from the data given for private genotypes within sites (above), there was strong structure, with CRN and HF differing from all sites for *A. tokioensis,* and CRN, AU, CW, and MTF differing from other sites for *A. agrestis*. A third measure, AMOVA, found that 30% of variation for *A. agrestis* (*F* = 0.30, *P* < 0.001) was accounted for by site, and for *A. tokioensis*, 17% for site (*F* = 0.17, *P* < 0.001). However, fully 94% (*F* = 0.05) of variation for *A*. *tokioensis* and 63% (*F* = 0.36) of variation for *A. agrestis* was partitioned by individual earthworm. This result would be explained if the earthworms are triploid.

**Table 5:** Population structure of *Amynthas tokioensis* and *Amynthas agrestis* across six sites in Vermont, USA, based on seven microsatellite markers. Given is Nei Unbiased Genetic Distance (D) above diagonal, and Rho, an F_*s*_*t* analogue for polyploid organisms, below diagonal. All pair-wise comparisons significant based on 999 permutations (Site codes: Commercial, Retail Nursery (CRN), National Audubon Society Nature Preserve (AU), Centennial Woods Natural Area (CW), Municipal Tree Farm (MTF), Home Garden (HG), Horticultural Research and Education Center of University of Vermont (HF)).

Population	CRN	AU	CW	MTF	HG	HF
*A. tokioensis*						
CRN	–	0.31	0.27	0.33	0.35	0.32
AU	0.61	–	0	0	0.07	0
CW	0.51	0	–	0	0	0
MTF	0.62	0	0.09	–	0.14	0.02
HG	0.66	0.46	0.65	0.67	–	0.04
HF	0.66	0	0.00	0.15	0.26	–
*A. agrestis*						
CRN	–	0.12	0.27	0.29	0.16	0.21
AU	0.21	–	0.23	0.28	0.18	0.05
CW	0.61	0.61	–	0.19	0.31	0.41
MTF	0.49	0.51	0.72	–	0.21	0.45
HG	0.27	0.32	0.71	0.46	–	0.32
HF	0.41	0.12	0.64	0.63	0.51	–

**Figure 4:**
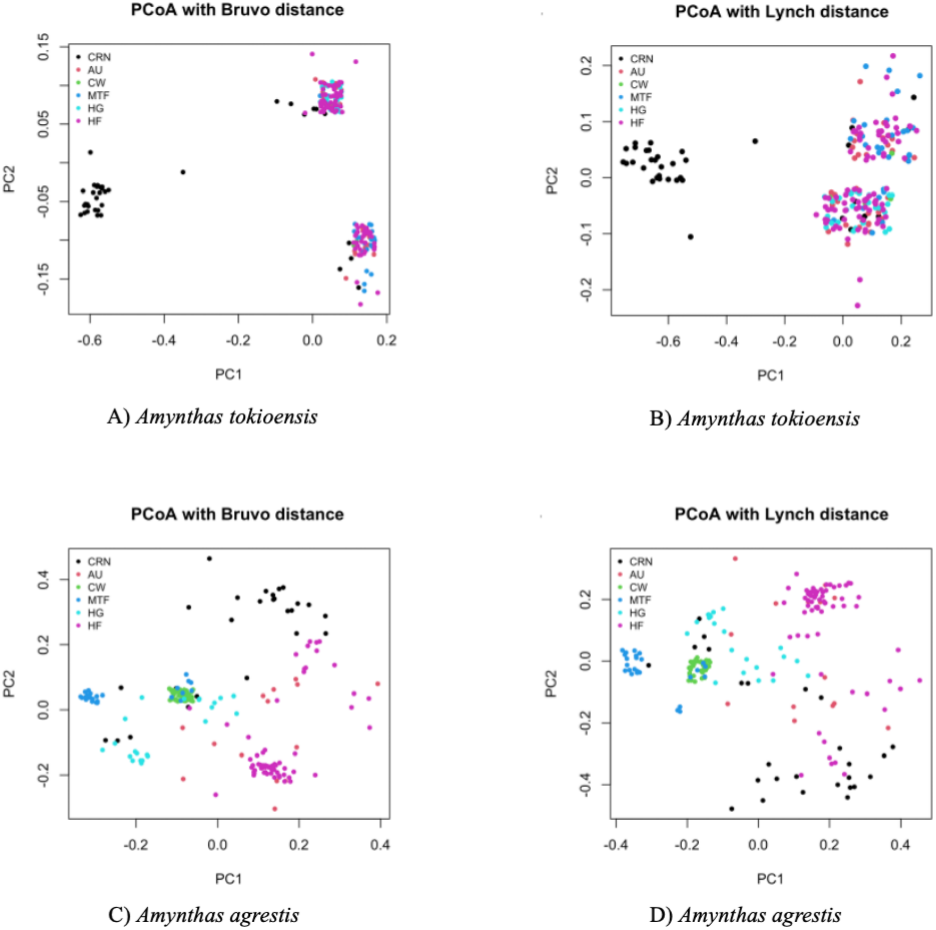
Principal Coordinate Analysis (PCA) with Bruvo’s distance (*A. tokioensis* (A), *A. agrestis* (C)) and Lynch’s distance (Lynch, 1990) (*A. tokioensis* (B), *A. agrestis* (D)). (A) PC1 explained 22% of the variance, PC2 explained 9% of the variance; (B) PC1 explained 24% of the variance, PC2 explained 7% of the variance. (C) PC1 explained 16% of the variance, PC2 explained 15% of the variance; (D) PC1 explained 20% of the variance, PC2 explained 18% of the variance (Site codes: Commercial, Retail Nursery (CRN), National Audubon Society Nature Preserve (AU), Centennial Woods Natural Area (CW), Municipal Tree Farm (MTF), Home Garden (HG), Horticultural Research and Education Center of University of Vermont (HF)).

Last, a Principal Coordinate Analysis (PCoA) placed each earthworm into a two-dimensional representation of the overall genetic diversity, with each earthworm scored to site (Fig. 4). *A. tokioensis* earthworms fell into three differentiated genotypic groups, with one group primarily from the CRN site, and the other two a mix of all the other sites. The pattern was quite different for *A. agrestis*, with greater genetic differentiation among the earthworms, but with several clusters by site. In summary, all measures indicate genetic structure among sites, but this structure is not simple (the origin of the earthworms by site is not simple), and most of the variation is partitioned to individual earthworms which is expected if they are primarily triploid and highly heterozygous.

## Discussion

*Amynthas* and other Pheretimoid earthworms are highly diverse (∼1,000 species in 12 genera) and distributed over a vast area through south, east, and southeast Asia, and into Australia. Only 16 of these species reported in North America are peregrine, meaning they are invasive over a large geographic region, even including oceanic islands (Chang et al., 2021). Although invasive *Amynthas* and related genera have been known in North America since at least 1867 (*A. agrestis* first seen in 1939 and *A. tokioensis* in 1947; (Reynolds, 2018)), they were seldom noticed until two decades ago when they became serious pests in forest and horticultural habitats (Frelich et al., 2006; Hendrix et al., 2006; Hendrix et al., 2008; Ferlian et al., 2018; Ferlian et al., 2020). In New England, USA, *Amynthas* have colonized habitats where other earthworms, typically about four to six species, are present (Görres, Bellitürk & Keller, 2014; McCay et al., 2017). These resident earthworms were themselves once non-native species, and thus the *Amynthas* are incomers that can reach very high population densities (up to 120 adults m^−1^ (Görres, Bellitürk & Melnichuk, 2016)) and can now dominate, and even eliminate, successful invaders that have become naturalized over the past three centuries (Chang et al., 2021).

The proposed characteristics that allow the *Amynthas* to enter complex, well established communities of other earthworms may be ecological such as broad diet (Zhang et al., 2010) and environmental tolerance, and ability of their eggs to survive harsh conditions (Görres, Bellitürk & Melnichuk, 2016) , have been studied, although such characters may be typical of other resident earthworms (Leirikh, Meshcheryakova & Berman, 2004). We used a different approach and examined the genetic characters of two invasive *Amynthas* species that may play an important role in dispersal, establishment, and success of these important invasive species: these were biparental or uniparental reproduction, and diploid vs. polyploidy systems. Also, we sought to determine the genetic diversity of the earthworms, and spatial patterns in their distribution.

Our findings are perplexing. Clonal reproduction was present for both species demonstrated by presence of groups of the earthworms with identical multi-locus genotypes. At some sites, all of the earthworms fell into a single genotype. This pattern was seen for both *Amynthas* species. Despite this clonal reproduction, there was a significant diversity of genotypes, fully 16 for *A. tokioensis* and 54 for *A. agrestis*, and many of these genotypes were found in only a single earthworm. Other parthenogenetic earthworms are also clonally diverse, but this diversity tends to be distributed among geographical sites (Novo et al., 2010; Fernández et al., 2011; Fernández et al., 2013; De Sosa et al., 2017), although high diversity of clones can be found very locally (Terhivuo & Saura, 2006). The high diversity of clonal genotypes at single sites is more suggestive of a sexually reproducing species. The number of microsatellite alleles for each locus and each species was also typical for biparental earthworm species (Keller et al., 2020). Thus, this high genetic diversity even with clonal reproduction begs the question if the earthworms sometimes reproduce sexually to generate that diversity of unique genotypes. Polyploidy was present, with all *A. tokioensis* triploid (highly unlikely to be currently sexual), but both triploid and diploid earthworms were found for *A*. *agrestis*. Male morphological parts (pores for exit of sperm) were not observed in *A*. *agrestis* and were present in only 19% of *A. tokioensis* earthworms. In subsequent years, though, we found male pores more common in both species (>10% for *A. tokioensis* and *A. agrestis*, personal observation). This polymorphism is the norm for these species. Pheretimoids have a wide range of morphologies from pure hermaphroditic morphs to those missing male function (Gates, 1954).

In conclusion, that it is likely the invasive *Amynthas* earthworms have mixed reproductive systems: both diploidy and triploidy within one species (*A*. *agrestis*), and parthenogenetic and possible sexual systems also within a species, at least in the recent past. The pattern seen would mean there was multiple origins for the many genetically-distinct clones observed. This result opens a window into the venerable debate on the adaptive significance of sex and the origin of polyploidy.

Sexual reproduction is present from protists to fungi to higher plants and all groups of animals, and the literature on the adaptive value of sex is vast. A simple Google Scholar search for “evolution of sex” yields 2.5 million hits, surely not all independent technical publications (useful reviews are (West, Lively & Read, 1999; Bell, 2019)). Earthworms, with their diverse reproductive systems, give an ideal model for studies of the evolution of sex (Introduction above). The advantages of sexual reproduction vs. parthenogenesis has long provoked contentious discussion, with a consensus view that generation of genetically variable offspring will aid in changing or spatially complex habitats. Jaenike and colleagues (Jaenike & Selander, 1979; Jaenike, Parker Jr & Selander, 1980; Jaenike, Ausubel & Grimaldi, 1982) reported this issue for earthworms, with both analytical models and studies of related parthenogenetic and sexual earthworm species. They concluded that sexual hermaphroditic earthworms are prone to a switch to parthenogenesis; that is, sexual reproduction is unstable for the hermaphrodites. Some benefit for sexual reproduction must therefore favor the sexual hermaphroditic forms, because most earthworm species seem to be sexual. They found that, contrary to the prevailing view of the benefit of sexual reproduction, the sexual forms were found in the more stable environments, and the parthenogenetic forms in the more challenging, unstable habitats. Their explanation is that if the ancestral condition is sex, and thus a high genetic diversity, the switch to parthenogenesis will result in many genetically-distinct clones. Selection would favor clones with an all-purpose phenotype, and thus a few clones would survive that can prosper in the unstable environments, such as the upper region of the soil (the epigeic life) with its threats from dry or very wet conditions and sudden changes in soil temperature. Note that parthenogenetic reproduction would also prevent any loss of such ideal genotypes by recombination typical of sexual reproduction.

The two *Amynthas* invasive species are epi-endogeic, and both over a single warm season and across years there were variable moisture conditions, and unpredictable changes in soil temperature, thus presenting a challenge for any introduced earthworm species (Snyder, Callaham & Hendrix, 2011; Görres et al., 2018; Nouri-Aiin & Görres, 2019). The great success of the *Amynthas* earthworms could be explained in part by a mixed reproductive system. Sexual reproduction could be episodic, or recently abandoned. The result would be substantial genetic diversity in the form of a large number of parthenogenetic clones. These events must have been recent because a substantial clonal diversity remains, as well as evidence for very recent sexual reproduction. This was the conclusion of a previous study on these *Amynthas* that used RAPD genetic markers that found both clones and singleton genotypes (Keller, Görres & Schall, 2017).

The two *Amynthas* species also differ in ploidy levels, with *A. tokioensis* all triploid and *A*. *agrestis* a mixture of diploid and triploid individuals. Biologists have been puzzled over the origin of polyploidy for a century, ever since technology allowed visualization of chromosome numbers. At the start, J.B.S. Haldane, one of the founders of evolutionary and population genetics, asked a curious question: Where did the extra chromosomes come from in polyploid species (Haldane, 1990). For plants, the main event seems to be hybridization between species or divergent genetic lines within a species, which leads to disruption of meiosis (allopolyploidy). Haldane proposed that this method would not be possible for animals, but he did not know then of the hybrid origin of polyploid animals, including some lizards (Cole et al., 2020). The alternative would be autopolyploidy within a single species in which some disruption of meiosis leads to the addition of an extra set of chromosomes. What would be any advantage of polyploidy? (Haldane, 1990) and others (Comai, 2005; Storchová et al., 2006; Otto, 2007; Gerstein & Otto, 2009) note that any deleterious mutations would be masked, with little opportunity for a triploid or higher ploidy level homozygous genotype for that mutation. But, over the generations, more mutations would accumulate, and individuals would benefit from a high level of heterosis. This view of the value of heterosis seems to be standard now (Comings & MacMurray, 1997; Comings & MacMurray, 2000; Comings, 1999). Jaenike & Selander (1979) proposed that during any switch from sexual to asexual reproduction in earthworms, there would be some mating between forms, and this would result in polyploidy offspring that would no longer be able to produce sexual offspring. Thus, if diploidy is the ancestral condition in earthworms, then the transition to triploidy in the *Amynthas* must be at its early stages. This transition may be evidenced also by different morphologies as is commonly observed for pheretimoid species (*e.g.*, Gates, 1954; Shen et al., 2011). Also, we doubt these earthworms are hybrids because the number of microsatellite alleles observed is typical for earthworms and the range in allele sizes shows no bimodal distribution expected from a hybrid. Again, this conclusion finds that the composite of reproductive systems in the two *Amynthas* species must be a result of recent or even ongoing events.

This study also provides insight into possible control measures to reduce spread of the pest earthworms. First, the largest number of genotypes for *A. agrestis* was found at a nursery (CRN) and a site adjacent to a tree farm (HF), so these may result in other sites being seeded with the earthworm eggs (resistant cocoons). Control measures like with entomopathogenic fungi (Nouri-Aiin & Görres, 2021) should be centered on horticultural nurseries. Second, if there are indeed all-purpose phenotypes among clones, then we would expect that the earthworms would be tolerant of any environmental threat. We are acutely aware that the microsatellite markers used here are assumed to be neutral, and useful for understanding reproductive systems, but do not tell anything directly about how phenotypes emerge. Thus, the missing information remains on specifically how the invasive earthworms can tolerate diverse and challenging environmental conditions.

## Conclusion

We have offered two large sets of microsatellite loci for two invasive species of earthworms for free use by researchers. Seven primer pairs per species exhibited consistent amplification with unambiguous scoring of alleles. Utilizing the selected microsatellite loci, we found clonal structure across all the sampling sites and also a high genetic diversity of clones which could be the result of multiple introductions and/or recent sexual reproduction. The worms were found to be both diploid and triploid.

These perplexing results (parthenogentic reproduction and large clonal diversity, and both ploidy levels) require further studies to answer whether these species of earthworms have both sexual and parthenogenetic reproduction.

##  Supplemental Information

10.7717/peerj.13622/supp-1 Supplemental Information 1 Number of genotypes, and measure of ploidy levels for the earthworm *Amynthas tokioensis* and *Amynthas agrestis* across five sites used in the study Genotype = code for distinct genotypes based on seven microsatellite loci, site code given in Table 1, possible ploidy levels is given as two (diploid) when one or two alleles are identified across all loci for an earthworm, or three (triploid) when three alleles are identified for at least one locus, and number of worms placed in clusters for each site.

10.7717/peerj.13622/supp-2 Supplemental Information 2 AMOVA table for *A. tokioensis* and [i]A. agrestis Used a ploidy independent Infinite Allele Model (corrected for unknown dosage of alleles), so the reported statistics are equivalent to Rho. Significance was tested using 999 permutations.

10.7717/peerj.13622/supp-3 Supplemental Information 3 Sequences for review (they all have accession numbers, but not published yet)

10.7717/peerj.13622/supp-4 Supplemental Information 4 Codes for US and Vermont maps

10.7717/peerj.13622/supp-5 Supplemental Information 5 Codes for PCoA analysis and figures

## Additional Information and Declarations

### DNA Deposition

The following information was supplied regarding the deposition of DNA sequences: The microsatellite loci sequences are available at GenBank: OM648919 to OM648932.

### Data Availability

The following information was supplied regarding data availability: The R codes for PCoA and maps are available in the Supplementary Files.
