# Supplementary material for: Genetic population structure and reproductive system of two invasive Asian earthworms, Amynthas tokioensis and Amynthas agrestis
Source: PeerJ. 2022 Jul 13;10:e13622. doi: 10.7717/peerj.13622 (PMC9288164; doi:10.7717/peerj.13622)
Supplement: Supplemental Information 1 — Genotype = code for distinct genotypes based on seven microsatellite loci, site code given in Table 1, possible ploidy levels is given as two (diploid) when one or two alleles are identified across all loci for an earthworm, or three (triploid) when three alleles are identified for at least one locus, and number of worms placed in clusters for each site. [file peerj-10-13622-s001.docx]

| Genotype | Population | Ploidy level | | # of earthworms | # of earthworms with male pore |
| --- | --- | --- | --- | --- | --- |
|  |  | Two alleles | Three alleles |  |  |
| *A. tokioensis* | | | | |  |
| G1 | CRN, AU, CW, MTF, HG, HF |  | 105 | 6, 15, 3, 8, 27, 46 | AU (3), MTF (3), HG (7) |
| G2 | CRN |  | 25 | 25 | CRN (7) |
| G3 | CRN, AU, CW, MTF, HF |  | 53 | 3, 16, 2, 15, 31 | CRN (1), AU (1), MTF (2) |
| G4 | CRN, MTF |  | 3 | 1, 2 |  |
| G5 | CRN |  | 1 | 1 |  |
| G6 | CRN, HF |  | 3 | 1, 2 |  |
| G7 | AU |  | 1 | 1 |  |
| G8 | AU |  | 1 | 1 | AU (1) |
| G9 | MTF |  | 4 | 4 |  |
| G10 | HF |  | 1 | 1 |  |
| G11 | HF |  | 1 | 1 |  |
| G12 | HF |  | 1 | 1 |  |
| G13 | HF |  | 2 | 2 |  |
| G14 | HF |  | 1 | 1 |  |
| *A. agrestis* | | | | |  |
| G1 | CRN |  | 1 | 1 |  |
| G2 | CRN |  | 1 | 1 |  |
| G3 | CRN |  | 1 | 1 |  |
| G4 | CRN |  | 1 | 1 |  |
| G5 | CRN |  | 1 | 1 |  |
| G6 | CRN |  | 1 | 1 |  |
| G7 | CRN |  | 1 | 1 |  |
| G8 | CRN |  | 1 | 1 |  |
| G9 | CRN |  | 1 | 1 |  |
| G10 | CRN |  | 1 | 1 |  |
| G11 | CRN |  | 1 | 1 |  |
| G12 | CRN |  | 1 | 1 |  |
| G13 | CRN |  | 4 | 4 |  |
| G14 | CRN |  | 1 | 1 |  |
| G15 | CRN |  | 1 | 1 |  |
| G16 | CRN |  | 1 | 1 |  |
| G17 | CRN |  | 1 | 1 |  |
| G18 | CRN |  | 1 | 1 |  |
| G19 | CRN |  | 1 | 1 |  |
| G20 | CRN |  | 1 | 1 |  |
| G21 | CRN |  | 1 | 1 |  |
| G22 | CRN |  | 1 | 1 |  |
| G23 | CRN |  | 1 | 1 |  |
| G24 | AU | 1 |  | 1 |  |
| G25 | AU | 1 |  | 1 |  |
| G26 | AU & HF | 16 |  | 3 & 13 |  |
| G27 | AU |  | 2 | 2 |  |
| G28 | AU |  | 1 | 1 |  |
| G29 | AU & HF | 5 |  | 1 & 4 |  |
| G30 | AU |  | 1 | 1 |  |
| G31 | AU |  | 1 | 1 |  |
| G32 | AU |  | 1 | 1 |  |
| G33 | AU |  | 1 | 1 |  |
| G34 | AU | 1 |  | 1 |  |
| G35 | CW & MTN |  | 50 | 45 & 5 |  |
| G36 | MTN |  | 17 | 17 |  |
| G37 | HG |  | 1 | 1 |  |
| G38 | HG |  | 11 | 11 |  |
| G39 | HG |  | 1 | 1 |  |
| G40 | HG & HF |  | 3 | 2 & 1 |  |
| G41 | HG |  | 1 | 1 |  |
| G42 | HG |  | 1 | 1 |  |
| G43 | HF |  | 1 | 1 |  |
| G44 | HF | 31 |  | 31 |  |
| G45 | HF | 1 |  | 1 |  |
| G46 | HF |  | 1 | 1 |  |
| G47 | HF |  | 1 | 1 |  |
| G48 | HF | 1 |  | 1 |  |
| G49 | HF |  | 1 | 1 |  |
| G50 | HF |  | 1 | 1 |  |
| G51 | HF |  | 1 | 1 |  |
| G52 | HF |  | 3 | 3 |  |
| G53 | HF |  | 3 | 3 |  |
| G54 | HF |  | 3 | 1 |  |
