# Supplementary material for: Genetic population structure and reproductive system of two invasive Asian earthworms, Amynthas tokioensis and Amynthas agrestis
Source: PeerJ. 2022 Jul 13;10:e13622. doi: 10.7717/peerj.13622 (PMC9288164; doi:10.7717/peerj.13622)
Supplement: Supplemental Information 2 — Used a ploidy independent Infinite Allele Model (corrected for unknown dosage of alleles), so the reported statistics are equivalent to Rho. Significance was tested using 999 permutations. [file peerj-10-13622-s002.docx]

| Source of variation | % VAR | Std.DEv. | F-value | P-value |
| --- | --- | --- | --- | --- |
| *A. tokioensis* | | | | |
| Within individual | 0.94 | 0.16 | 0.05 | - |
| Among individual | -0.12 | 0.16 | -0.14 | 1.000 |
| Among population | 0.17 | 0.04 | 0.17 | 0.001 |
| *A. agrestis* | | | | |
| Within individual | 0.63 | 0.17 | 0.36 | - |
| Among individual | 0.06 | 0.16 | 0.08 | 0.001 |
| Among population | 0.30 | 0.07 | 0.30 | 0.001 |
