# Supplementary material for: Genetic population structure and reproductive system of two invasive Asian earthworms, Amynthas tokioensis and Amynthas agrestis
Source: PeerJ. 2022 Jul 13;10:e13622. doi: 10.7717/peerj.13622 (PMC9288164; doi:10.7717/peerj.13622)
Supplement: Supplemental Information 3 [file peerj-10-13622-s003.docx]

***Amynthas tokioensis***

**>Contig906 16 reads, 304 bases**

**TM right:** 60.262

**Right Primer:** CAAAGGAAGGAAAGACGGTAACC

**TM left:** 60.138

**Left Primer:** GAGTGCAGTCCATTCCATTAGTG

**Product Size:** 206

**Repeat sequence: AAGT**

TACCGTAAGCACCATCGCTACATCAATCGAACATTTTAGCAACTTGACGCGACAGAAATGGCAAAATTCGAGTGCAGTCCATTCCATTAGTGAATAGTCGAGTGCTCTCTGTACAACTGTAAATCGTAGCAAGTAAGTAAGTAAGTAAGTAAGTAAGTAAGTAAGTAAGTAAGTAAGTAAGTAAGTAAGTAAGTAAGAGAGCTGAAGAGTTTTCGCGCCTTCAATTTTTCACGTCATCGGTCGAATGAGGAGGGTTACCGTCTTTCCTTCCTTTGCCAGGTGAGCGCAAATTCGCAGGTAAGTC

**>Contig8188 18 reads, 503 bases**

**TM right: 59.765**

**Right Primer: CATGCCTAGAGAGAATAAGCAGC**

**TM left: 59.451**

**Left Primer: TGAGCATGAGTTGAGAGTTTGAG**

**Product Size: 229**

**AAGT- 25**

ACGATCGGTAGTTACCTGATTAACTGATAATTAACCGAGATTAACTGATTGTTTCCAGTAAAATGAGTTTATGAGTTAACTCACATTATTGACGAGTTTGAGTTAACTCATTCAAAACAGAGTGAATTGAGTTTGAGTTTAAACTCATTTAAA**TGAGCATGAGTTGAGAGTTTGAG**TTAACTCATTTATTGTATGAGTTTTCCCAACACTGT**AAGTAAGTAAGTAAGTAAGTAAGTAAGTAAGTAAGTAAGTAAGTAAGTAAGTAAGTAAGTAAGTAAGTAAGTAAGTAAGTAAGTAAGTAAGTAAGTAAGT**AAAATCTCGTAGATTTATACAGCGCTTCGAAAAGTAGTACAGTAGTA**GCTGCTTATTCTCTCTAGGCATGCATGATATCTGTA**TAGCTCGTATCTCAACAACAGAAACCAAACTAAAAAACGTGCATCGGCACAGCGAGTCATACTGCAATGGAATGACATCATTTCCAAAAACTAATCACATCATTTGTC

**>Contig943 28 reads, 383 bases**

**TM right:** 60.507

**Right Primer:** CTGTTAAGTGTTCAGTGTTCCCG

**TM left:** 60.139

**Left Primer:** TTCGTATTCAAGAGTTTCGCGAG

**Product Size:** 295

**Repeat sequence: AAGT**

ACTTAACATGTTCTGTCTATTAGGGGACTGCCAAGAGTTGATTTGTTAATGT**TTCGTATTCAAGAGTTTCGCGAG**AAAACCGTCCTCGAAACGCTTATGCAGAGAGCGTGAACAGGCATTTTGATTTTGATCATAGATTATAAGTAATAAGCCTATATGGATAAATCTATACAATTTCATTAGGCTTCGAACTTTGTTATAGAAAGACTTT**AAGTAAGTAAGTAAGCAAGTAAGTAAGTAAGTAAGTAAGTAAGTAAGTAAGTAAGTAAGTAAGTAAGTAAGT**GAACGTTAAGATTAAAGTGGTAACTCGTCTCAAAAAGACTG**CGGGAACACTGAACACTTAACAG**AAACATAAACATAATTAATTAACCTAGATAGCTAGT

**>Contig984 57 reads, 348 bases**

**TM right:** 60.138

**Right Primer:** ATGCAATGTACTGAAGTGCTAGC

**TM left:** 60.568

**Left Primer:** AATGAATTTGTGTCGTCTGTCCG

**Product Size:** 178

**Repeat sequence: AAGT**

CACTCCTCGAAAACAGGGCCTTACTGGTGC**AATGAATTTGTGTCGTCTGTCCG**TCACGAACCCTTGTTGCTACCTTGTACAGCTGTACTGGTGGTCGT**AAGTAAGTAAGTAAGTAAGTAAGTAAGTAAGTAAGTAAGTAAGT**AAAATCTCTACAGCAACTTAATAGCATTTTCTTACCTTCATTG**GCTAGCACTTCAGTACATTGCAT**TCGTGTCTGTGTATGTTGTGCTTGTTTATAGACGCGTCCGTATCAAAGACGTGTCTGCTTAAGCTGACTGTGATAAGCCAGCAATAACCATCGGTTAATTAAAATCTCTCAACGCGGCACGTTATCATGCTGGCTGCGTC

**>Contig1011 60 reads, 425 bases**

**TM right:** 59.829

**Right Primer:** CTACTGCGGTACTTCTTTCCAAG

**TM left:** 59.956

**Left Primer:** GAATTCTTAGTCGCGTACAGTCC

**Product Size:** 229

**Repeat sequence: AAGT**

AACCGCTGAAGTAATCCTGTCGCATAATAATTAACTTAATCAAATTAATCATCCCTTCG**GAATTCTTAGTCGCGTACAGTCC**GTGATATTTTAATCTCGTGCGACGATTGCACACTGCACTCAAATTCCACGACAAAGCCAAACAGAAATTTGAAACCCAGATCAATATAGTATAAGCAAGCAAGC**AAGTAAGTAAGTAAGTAAGTAAGTAAGTAAGTAAGTAAGTAAGTAAGTAAGT**AAGAATCTCGTGAATTTCTTTAGCGCC**CTTGGAAAGAAGTACCGCAGTA**GCCCCTCATTCCTGAGCGCTTAAATAAAAAAATATAATAAACAGTTAATACATAATTTCTGGCCTCTCAACAAAAGTTTGGACAGAATCGTTAAATCGGAACAAGTTTCTCATAACTTCGGATCACATTAAACCAGTT

**>Contig1053 11 reads, 355 bases**

**TM right:** 60.264

**Right Primer:** AACCTCTTCAAGACCTATGGACC

**TM left:** 61.199

**Left Primer:** TTGGTGATCGCTACCAATGAGAG

**Product Size:** 214

**Repeat sequence: ATC**

CTCTTGATCGAAATTGACCTATTGGCATCACAAGTT**TTGGTGATCGCTACCAATGAGAG**GTTAATGCATTCTTGTAGTCTACGTAACTACACATCATATTTTTCGCAAC**ATCATCATCATCATCATCATCATCATCATCATCATCATCATCATCATCATCATCATCATCATCATC**GCATAGCCATCTGGGGCCGTTGCGTTGCTCCATGTCCCATGTTGATCGCGTC**GGTCCATAGGTCTTGAAGAGGTT**CGTCGGAGTCGTTCTTCAGCTGGTCCAGCCAGGTTTTCCTTGGACGACCGCGAGGTCTCTTCCATCCAGGGTGAGGCGGTCGGGTCTTCGCTGAGTGGACTGGGT

**>Contig1176 52 reads, 387 bases**

**TM right:** 59.833

**Right Primer:** GCATTAACTGTGTGACATGCTTG

**TM left:** 59.539

**Left Primer:** CAGTTAAAGATTTGTTGACCTGCC

**Product Size:** 319

**Repeat sequence: ACAT**

CTTTAATCTTGTGTCAGTAAAGTTTCAAGATTTGT**CAGTTAAAGATTTGTTGACCTGCC**AATTTAATTTTCTGACCCCTGTCATGGGCGCCAAAATACCAAAAGTACATACACCCCCAAAAGGCACTAAATACAAGCAGCAGCCAAAGCAACAAATAAGAAACTATTCGTTACACGCATATATACAT**ACATACATACATACATACATACATACATACATACATACATACATACATACATACAAGCATACAT**ACGGAGTACAAGGCACTGATGTTTCAAGCATCCATTGCTTATCCAGATCAACAGAATTGCAGCTATACTAGTTTTTTTTA**CAAGCATGTCACACAGTTAATGC**CAAATTTCAAATGATTTACATGTTAAATGAGTC

***Amynthas agrestis***

**>Contig5016 28 reads, 354 bases**

**TM right:** 60.137

**Right Primer:** ATAAATGACCCTGCACGAATGAC

**TM left:** 58.051

**Left Primer:** AATACATTACTTTCGTCGCTGAC

**Product Size: 302**

**Repeat sequence: AAGT**

CTCAAAAGAGGGGTCCATCA**AATACATTACTTTCGTCGCTGAC**GCAATTTTGCGCCACCTGGCGAATATCAGCGAAACGCTTTCGCTACGTACTAAAAAATTATTGTCGGCC**AAATGCTAAGTAAGTAAGTAAGTAAGTAAGTAAGTAAGTAAGTAAGTAAGTAAGTAAGTAAGTAAGTAAGTAAGTAAGTAAGT**AAGACTCTCGTTAATTTCTATAGCGCCTTAAAAAGAAATACCGCAGTAACTCCTTAGTCATAGGTGCTTAGTCCTAATGTGAAATAGCTATAGCCAGAGGTGGG**GTCATTCGTGCAGGGTCATTTA**TTCCAAAGGGTCATTGTGGTTGGGACAGTACAG

**>Contig628 25 reads, 240 bases**

**TM right: 59.333**

**Right Primer: TGACTCTCTAATGCGCTTGTTAG**

**TM left: 60.75**

**Left Primer: GTAGAACGACGGACTCTGAGATC**

**Product Size: 195**

**Repeat sequence: ACAT**

ACTTGTCCGGATAGTTGGGACTCGAGATGTATCCGTGGTC**GTAGAACGACGGACTCTGAGATC**CAGGACATCCGTTCTGGATCAGGAGAACT**ACATACACACATACACACATACATACATACATACATACATACATACATACATACATACATACATACATACATACATACATACATACATACAT**ACACCAGCGCCTGTTCAATAATTAATTG**CTAACAAGCGCATTAGAGAGTCA**ATTGT

**>Contig6034 27 reads, 315 bases**

**TM right:** 60.263

**Right Primer:** TGGATGAAAGGGAATGTTCGTTG

**TM left:** 60.014

**Left Primer:** TAAACTGAAGAACTGCATCCACG

**Product Size: 290**

**Repeat sequence: ACAG**

**CTGCAGGTAAACTGAAGAACTGCATCCACGCATGTTCCATTGTTAAAACAGGGATTGCTTACACAATCGTCTATATTCTGCTCACAGTTGAAGCCGTCAAATCCTGTAACAGTACACAGAAACATAGTTAAGCAATTTACATGATATACCCTTGACATGAGAGACAGACAGACAGACAGACAGACAGACAGACAGACAGACAGACAGACAGACAGACAGACAGACAGACAGACAGACAGAGAACTATTTATAAAGCTCTACATAATTTTCTGTTCAACGAACATTCCCTTTCATCCAGTATGATTAAAATCAGTC**

**>Contig652 11 reads, 385 bases**

**TM right: 59.828**

**Right Primer: CTCTTGGTGTGATCATGTGACTC**

**TM left: 59.645**

**Left Primer: ACTACGTGATCAAACCTTATCGC**

**Product Size: 290**

**TCTA (AGAT)- 26**

CACGGAGCTGGAATACGCTTCCGACTTC**ACTACGTGATCAAACCTTATCGC**GTCCAGTATTTCTGAACAAACTCAAGATGCTATTATTTTGACTAGCAGCAGCGGCGCACTTGCAAAAGTAGCGCCTTAGAAATTACTT**TCTATCTATCTATCTATCTATCTATCTATCTATCTATCTATCTATCTATCTATCTATCTATCTATCTATCTATCTATCTATCTATCTATCTATCTATCTATCTA**TAGGTGATCCGCTCCAGTACTTTCAGCCAGTGGAGCATGTCTTTAAGGAGCG**GAGTCACATGATCACACCAAGAG**GATCGGAAGATCAGGCATGCTGAGGCATTCAGCACCATCTGTAGGCGGCTTAGCTGATGATCCGGTA

**>Contig13732 16 reads, 286 bases**

CTGTGATGCAA**AGCAGTTTGTTAGACCACATAATTC**AAAAACCGACCATCTGGACCCTTTTCATGTTCAGGGCCACATTTTA**TCTATCTATCTATCTATCTATCTATCTATCTATCTATCTATCTATCTATCTATCTATCTATCTATCTATCTA**TGATGAAAATTTGCGAGTATGAAGATGATTTTCAAACTAAAATGACATCCTGAGAACTAAATGAGACTAAAAAAGTTT**CGTGATCATTGACATCTGCAAGA**CATCATTGCCTACACTGTCTCTCAATGGCAG

>**Contig3786** 19 reads, 290 bases

ACTACTACAATG**GCGCTAAAGAAGTCTACTGCTAC**TGCTGCTGCTGCTGCTGCTGCTACTGCTGCTACTGCTACTGCTACTGCTACTACTACTGCT**ACTACTACTACTACTACTACTACTACTACTACTACTACTACTACTACTACTACTACTACTACTACTACTACTACTACTACTACT**AGTATCTAAAGAACTGCAAAATG**CACAGTACAATGTAGTAGTGCAG**ATACCAATTTCTGATGAGAAACCAGTAATTTCAGATGTCCAATCACTTTTTGACCTAAACAGTC

>**Contig9389** 12 reads, 232 bases

TACTTCTGAATACATGAATGGGTTTGTTGTTGTTGTTCATCATCCATTATCATCATGTTCATCTTCTTCTTCTACTACTACTACTACTACTACTACTACTACTACTACTACTACTACTACTACTACTACTACTACTACTACTACTACTACTACTACTTCTGCTTCTTCTTCGTTTTTTCTTCTTCTTCCAGTTCTTTAATCGTATTCTTGTGAGATCCGTTCGCTGGAAGTG
